# Vein network redundancy and mechanical resistance mitigate gas exchange losses under simulated herbivory in desert plants

**DOI:** 10.1093/aobpla/plad002

**Published:** 2023-01-24

**Authors:** Miguel A Duarte, Sabrina Woo, Kevin Hultine, Benjamin Blonder, Luiza Maria T Aparecido

**Affiliations:** School of Life Sciences, Arizona State University, 427 E Tyler Mall, Tempe, AZ 85281, USA; School of Life Sciences, Arizona State University, 427 E Tyler Mall, Tempe, AZ 85281, USA; Department of Research, Conservation and Collections, Desert Botanical Garden, 1201 N. Galvin Parkway, Phoenix, AZ 85008, USA; School of Life Sciences, Arizona State University, 427 E Tyler Mall, Tempe, AZ 85281, USA; Department of Environmental Science, Policy, and Management, University of California Berkeley, 120 Mulford Hall, Berkeley, CA 94720, USA; School of Life Sciences, Arizona State University, 427 E Tyler Mall, Tempe, AZ 85281, USA; School of Earth and Space Exploration, Arizona State University, 781 Terrace Mall, Tempe, AZ 85287, USA

**Keywords:** Gas exchange, herbivory, leaf traits, leaf venation, Sonoran Desert

## Abstract

Herbivory can impact gas exchange, but the causes of interspecific variation in response remain poorly understood. We aimed to determine (1) what effects does experimental herbivory damage to leaf midveins have on leaf gas exchange and, (2) whether changes in leaf gas exchange after damage was predicted by leaf mechanical or venation traits. We hypothesized that herbivory-driven impacts on leaf gas exchange would be mediated by (1a/1b) venation networks, either by more vein resistance, or possibly trading off with other structural defenses; (2a/2b) or more reticulation (resilience, providing more alternate flow pathways after damage) or less reticulation (sectoriality, preventing spread of reduced functionality after damage). We simulated herbivory by damaging the midveins of four leaves from each of nine Sonoran Desert species. We then measured the percent change in photosynthesis (*ΔAn%*), transpiration (*ΔEt%*) and stomatal conductance (*Δgsw%*) between treated and control leaves. We assessed the relationship of each with leaf venation traits and other mechanical traits. *ΔAn%* varied between +10 % and −55%, similar to *ΔEt%* (+27%, −54%) and *Δgsw%* (+36%, −53%). There was no tradeoff between venation and other structural defenses. Increased damage resilience (reduced *ΔAn%*, *ΔEt%*, *Δgsw%*) was marginally associated with lower force-to-tear (*P* < 0.05), and higher minor vein density (*P* < 0.10) but not major vein density or reticulation. Leaf venation networks may thus partially mitigate the response of gas exchange to herbivory and other types of vein damage through either resistance or resilience.

## Teaser

How do vein networks influence plant resistance or resilience to herbivory? While vein networks are widely understood to influence rates of resource transport in undamaged leaves, they may also promote resistance to damage or resilience of resource transport after damage. We tested this hypothesis via experimental herbivory and gas exchange measurements on nine Sonoran Desert plant species. We found that vein network architecture and mechanical traits do have an effect on gas exchange responses to experimental herbivory. Leaf venation networks thus partially mitigate the response of gas exchange to vein damage through either resistance or resilience.

## Introduction

Herbivores consume large fractions of plants’ annual primary productivity (Coley *et al.* 1985). The impacts of herbivory include both the direct loss of carbon and soil nutrient investment in leaf tissue, indirect loss of future carbon gain via lost photosynthesis and/or reduced leaf lifespan (Crawley 1983; Nabity *et al.* 2009), as well as the reduction of transpiration and stomatal conductance for leaf cooling (Havko *et al.* 2020). Some plant species can be less affected by herbivory than others due to variation in their ecological strategies. Examples of these strategies include *tolerance*, by growing compensatory tissue; *resistance*, by evolving traits that reduce palatability and consumption by herbivores; and *avoidance*, by decreasing their accessibility to herbivores spatially or temporally. The tolerance and resistance strategies have been broadly studied from the perspective of widely-recognized defenses, which include secondary chemistry, physical traits (such as greater mechanical strength), and mutualisms (Bronstein *et al.* 2006).

Leaf veins transport resources including water and photosynthate and also provide mechanical strength to the leaf through their lignified tissues. Venation networks could thus influence herbivory responses according to several hypotheses, either through allocation to veins as a form of herbivory resistance (Hypotheses 1a/1b) or patterning of veins via reticulation as a form of herbivory tolerance (Hypotheses 2a/2b).

To explore herbivory resistance, Hypothesis 1a considers that greater investment in veins (i.e. higher vein length density or vein volume density) could increase leaf structural defenses, increasing herbivory resistance. Organic chemical defenses, structural compounds and/or secondary compounds, reduce the incentive for herbivores to feed on plants (War *et al.* 2012) and plants allocating more resources toward secondary compounds are more unpalatable to herbivores than other species that do not produce these compounds (Augustine and McNaughton 1998). Plants have developed strategies and defenses to counteract the effects from different types of herbivory attacks (Johnson 2011). For instance, stiffer and stronger leaves are more difficult for chewing insects, such as leafcutter ants or caterpillars, to damage (Caldwell *et al.* 2016). This hypothesis is consistent with a range of empirical studies showing linkages between veins and mechanical properties (Niinemets *et al.* 2007; Kawai and Okada 2016; Blonder *et al.* 2018; Hua *et al.* 2020), linkages between veins and secondary defenses (Nabity *et al.* 2009), as well as various modelling studies that reach similar conclusions (Lin *et al.* 2020; Ronellenfitsch 2021). However, some studies have found decoupling between hydraulic and mechanical functions, possibly mediated by variation in leaf tissue density (Méndez-Alonzo *et al.* 2019) or presence of bundle sheath extensions (Ohtsuka *et al.* 2018).

Also to explore herbivory resistance, Hypothesis 1b considers that greater investment in veins could drive allocation trade-offs with other defense strategies, either decreasing herbivory resistance or leading to no net effect on herbivory resistance (opposite prediction to Hypothesis 1a). This hypothesis is consistent with the theory that defenses are costly, reducing the fitness relative to undefended plants when predators are not present and beneficial, enhancing fitness relative to undefended plants when predators are present. (Zangerl and Bazzaz 1992; Stamp 2003). High allocation of resources toward a single trait have been reported to result in greater negative effects on other defensive compound synthesis (Züst and Agrawal 2017).

To explore herbivory tolerance, Hypothesis 2a considers that greater venation network reticulation (i.e. higher loopiness) could provide redundant flow pathways and higher resilience for resource flow under disruption, increasing herbivory resilience. This hypothesis is consistent with theory for optimal network architecture under damage (Corson 2010; Katifori *et al.* 2010; Ronellenfitsch and Katifori 2019). It is also consistent with an empirical study showing improved resilience of hydraulic conductivity after experimental wounding in palmate vs pinnately veined species (Sack *et al.* 2008). Reticulation in other vein orders (besides major veins) may also allow for flow despite damage. Reticulation also could be relevant for herbivore defense in latex-producing species, which have laticifers whose paths follow subsets of the venation network. Chewing insects sometimes attempt to disrupt latex defenses by severing laticifers, a process that presumably is less successful when reticulation is high (Delaney and Higley 2006; Agrawal and Konno 2009). This hypothesis has not been extensively assessed in species with a wide range of leaf network architectures.

Further on herbivory tolerance, Hypothesis 2b considers that greater reticulation reduces network sectoriality, enabling the spread of embolism or leaf diseases introduced by herbivores, decreasing herbivory resilience (opposite prediction of Hypothesis 2a). However, this hypothesis has not been explored for leaf venation responses to herbivory, though it has been explored in the context of stem xylem responses to embolism (Zanne *et al.* 2006; McElrone *et al.* 2021; Wason *et al.* 2021), where it may provide resilience under environmental stress (Salguero-Gómez and Casper 2011). It is also consistent with theory showing disease spread is slower in more modular networks (Salathé and Jones 2010).

Here, we focus primarily on testing these contrasting hypotheses in Sonoran Desert plants with a herbivory tolerance strategy (i.e. not annuals, or drought-deciduous taxa; Marchin *et al.* 2010). Desert plants experience extreme heat and low water availability, leading to potentially greater consequences of herbivory for plant performance than in other biomes (Shreve and Wiggins 1964; Orians and Solbrig 1977; Villar and Merino 2001).

Leaf tissues in desert plants are especially valuable because plants that grow in stressful environments usually cannot store enough labile carbon to regularly rebuild damaged tissues following herbivory, as adaptations to drought and heat are already costly (Solbrig and Orians 1977; Wright *et al.* 2004; Smith *et al.* 2012; Aparecido *et al.* 2020). However, combined plant responses to herbivory and other environmental stressors have been under-investigated in desert plant taxa. Likewise, information of how leaf venation networks are correlated with herbivory tolerance/ damage have been largely unexplored, particularly in desert plants (Barreto *et al.* 2021).

Based on the hypotheses described, we specifically ask: 1) what effects does experimental damage (simulated herbivory) to midveins have on the resilience of post-damage leaf gas exchange (photosynthesis, transpiration and stomatal conductance)?; 2) are mechanical traits associated with venation traits, and 3) is post-damage leaf gas exchange best predicted by mechanical traits (e.g. force-to-tear, leaf dry matter content), vein investment (vein density), and/or vein reticulation (mean spanning tree ratio, hereafter MST ratio)? We explored these hypotheses and questions when considering veins of all size classes as well as only large size classes, as vein functioning may differ by size (Sack and Scoffoni 2013; Blonder *et al.* 2020).

## Materials and Methods

### Study site and sampling

**Figure 1.**
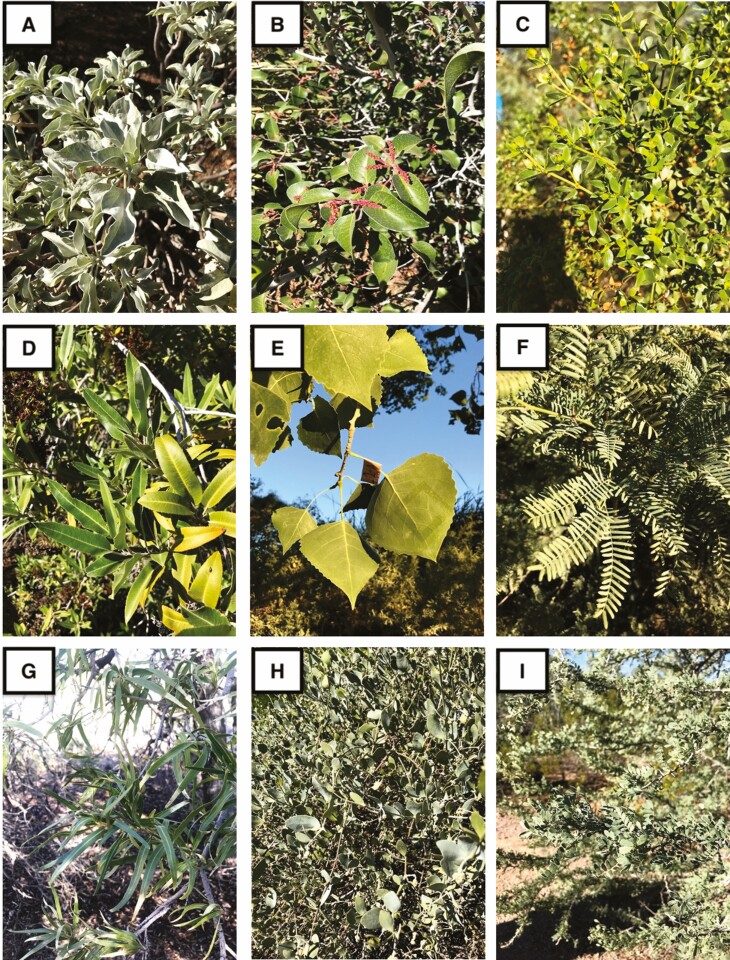
Representative Sonoran desert species sampled in the present study. Plant species: **A**) *Encelia farinosa*; **B**) *Rhus ovata*; **C**) *Larrea tridentata*; **D**) *Vauquelinia californica*; **E**) *Populus fremontii*; **F**) *Prosopis velutina*; **G**) *Chilopsis linearis*; **H**) *Simmondsia chinensis*; **I**) *Olneya tesota*.

This study included nine species of Sonoran Desert plant taxa that had diverse leaf traits and a common C3 photosynthetic pathway (Table 1; Fig. 1), enabling control of water/carbon use strategy. Individuals were studied at the Desert Botanical Garden in Phoenix, Arizona, USA (33.4618° N, 111.9446° W) in June 2018. We selected individuals in low-irrigation, semi-natural environments away from public trails and with no signs of thermal damage. We chose two healthy and mature individuals (trees/shrubs) for each species (*n* = 18 plants).

**Table 1.** List of plant species. Photos of each species are shown in Fig. 1. Leaf size corresponds to the entire leaf area, including all leaflets (*Larrea tridentata* = 2 leaflets; *Olneya tesota* = 12-16 leaflets; *Prosopis velutina* = 14-18 leaflets).

Species	Family	Common name	Growth form	Leaf size (cm^2^)
*Chilopsis linearis* (Cav.)	Bignoniaceae	Desert willow	Tree	2.85 ± 0.60
*Encelia farinosa* A. Gray ex Torr.	Asteraceae	Brittlebush	Shrub	7.41 ± 3.34
*Larrea tridentata* (DC.) Coville	Zygophyllaceae	Creosote	Shrub	0.20 ± 0.50
*Olneya tesota* A. Gray.	Fabaceae	Desert Ironwood	Tree	6.45 ± 0.66
*Populus fremontii* S. Watson	Salicaceae	Cottonwood	Tree	45.46 ± 5.14
*Prosopis velutina* (Wooton) Sarg.	Fabaceae	Mesquite	Tree	10.57 ± 2.72
*Rhus ovata* S. Watson	Anacardiaceae	Sugar sumac	Shrub	20.97 ± 5.51
*Simmondsia chinensis* (Link) C.K. Schneid.	Simmondsiaceae	Jojoba	Shrub	6.65 ± 2.08
*Vauquelinia californica* (Torr.) Sarg.	Rosaceae	Rosewood	Tree	8.91 ± 1.12

### Experimental treatments

To investigate the effects of herbivory damage on leaf gas exchange, a control and a treatment were applied to a set of healthy, mature, and sunlit leaves of each individual. The simulated herbivory treatment **[see ****Supporting Information—Fig. S1****]** consisted of piercing the primary vein(s) one-third of the primary vein length, above the petiole insertion using a thick Glovers needle **[see ****Supporting Information—Fig. S1A**]. For compound leaves, around 70 % of the leaflets were punctured through the middle of the primary vein **[see ****Supporting Information—Fig. S1B**]. Considering the possibility that the punctures would cause extreme damage to one or more leaflets if the needle missed the midvein, thus compromising further leaf functioning, we left some leaflets whole. These leaflets would then represent the undamaged portion of the treated leaves (similar to the other two-thirds of the single leaf). This treatment partially simulates piercing/ chewing herbivory via direct mechanical damage (**[see ****Supporting Information—Fig. S2****]**; Dussourd and Eisner 1987; Delaney and Higley 2006), but omits the additional effects of chemical signalling/sensing that would occur with real herbivores (Baldwin 1990).

From the two individuals of each species, two sun-exposed branches were selected for each plant for this study, from which four leaves or set of leaves (e.g. when leaves were too small or compound) with no apparent leaf damage were selected. The study thus included a total of 9 species × 2 individuals × 2 branches × 2 treatments × 2 leaves = 144 total leaves; with exception of one *Larrea tridentata* branch that was not treated properly and thus both branches excluded (4 leaves) from the experimental design, and one *Encelia farinosa* leaf that fell before being measured, totalling 139 leaves for the entire gas exchange dataset.

### Gas exchange measurements

Net photosynthesis (*An*; μmol CO_2_ m^−2^ s^−1^), transpiration (*Et*; mol H_2_O m^−2^ s^−1^) and stomatal conductance (*gsw*; mol H_2_O m^−2^ s^−1^) measurements were conducted on 21 and 22 June 2018 on control leaves and on leaves that received simulated herbivory damage at 12PM-2PM the day prior (18–20 h before gas exchange measurements). Puncturing the leaves within 24 h guaranteed that leaves would not die before gas exchange measurements could be taken. Measurements were conducted in the morning period (8AM–1PM) using a portable photosynthesis system (LI6800, LI-COR Inc., Lincoln, NE; **[see ****Supporting Information—Fig. S3****]**) with fixed flow rate = 600 μmol s^−1^, carbon dioxide concentration (Ca) = 400 μmol mol^−1^, and light intensity (photosynthetically active radiation, PAR) set at 1500 µmol m^−^² s⁻¹. Ambient environmental conditions for the study period had, on average, air temperature ranging between 32 and 41 °C (early morning until midday), relative humidity between 10 and 21 %, vapor pressure deficit between 4 and 7 kPa, and PAR approximately between 1000 and 2000 µmol m⁻² s⁻¹ (Aparecido *et al.* 2020). Cuvette conditions were set to track ambient conditions (i.e. air temperature and relative humidity).

Measurements were logged when *An*, *Et*, and ΔCO_2_ and ΔH_2_O concentrations (i.e. difference between CO_2_, H_2_O supplied by the system (reference) and CO_2_ assimilated, H_2_O released by the leaf inside the chamber (sample)) were stable (i.e. through real-time graphing of gas exchange responses and when ΔCO_2_ and ΔH_2_O concentrations were within standard deviation ± 0.1). Additionally, a second measurement was logged after 1 min of the first measurement. The area enclosed by the chamber was marked **[see ****Supporting Information—Fig. S3B****]** and later was used to correct gas exchange values that were originally estimated based on a full chamber area coverage (9 cm^2^). The chamber enclosed the leaf without including the vein damage when leaves were longer than 3 cm **[see ****Supporting Information—Fig. S3B****]**; however, this was not possible with smaller leaves/leaflets (i.e. *Larrea tridentata*, *Prosopis velutina*, and *Olneya tesota*; **[see ****Supporting Information—Fig. S1B****]**. *Populus fremontii* was the only species to cover the entire chamber. We were not able to standardize the portion of the leaf included in the chamber.

### Leaf traits

Leaf dry matter content (LDMC; g/g) estimates **[see ****Supporting Information—Appendix S3, Data S2****]** were obtained from leaves with petioles still attached since most leaves had small or non-distinguishable petioles (e.g. no clear separation between petiole and leaf lamina). Leaves were sampled from three plants, including the plants sampled in the present study, due to a more extensive data collection from a related study done by Aparecido *et al.* (2020). All leaves collected for leaf mechanical and venation trait determination were mature, unherbivorized and collected only if not wilted or thermally damaged.

As described by Pérez-Harguindeguy *et al.* (2013), leaves were stored and rehydrated, for at least 24 h, by wrapping them in a wet paper towel, storing them in an impermeable plastic bag, and keeping them cool and in the dark within a refrigerator until their wet mass was taken using a microbalance in the next 48 hours. The samples were then dried for 48 h at 60 °C in a drying oven and then their dry mass was taken. LDMC was calculated by finding the ratio between the leaves’ dry mass divided by wet mass (Pérez-Harguindeguy *et al.* 2013). Scans were taken of each fresh leaf to retrieve a one-sided area using ImageJ (Schneider 2012; Rueden 2017). Images were also taken of acrylic peels from both leaf surfaces to determine stomatal occurrence following methods from Aparecido *et al.* (2017). These images were used to correct gas exchange calculations, where the portable photosynthesis system’s stomatal ratio parameter was set *K* = 0.5 for hypostomatous leaves and *K* = 1.0 for amphistomatous leaves. Leaf mass per area (LMA) was also calculated but results using this trait were similar for LDMC and are not presented, but raw data are available in the **Supporting Information**.

Using the same protocol for selecting leaves for gas exchange, new fully grown leaves were selected from the same individuals to estimate *force-to-tear* (N/mm; **[see ****Supporting Information—Data S3****]**). A tensometer was used to tear each sample by placing and tightening a clamp on each end of the leaf, keeping it taut **[see ****Supporting Information—Fig. S4****]**. A gear was then turned by continuously cranking a lever, slowly pulling one clamp away from another until the leaf was torn, while a force gauge attached to the tensometer was used to find the maximum force recorded before tearing occurred. Larger leaves were cut to 3.81 cm (or 1 1/2 inch) in length and 0.60 cm in width to standardize leaf size across species. Leaves or leaflets smaller in length/width were not cut. Leaf fractions avoided the midvein as much as possible. For leaves and leaflets already smaller than these dimensions (*Prosopis velutina*, *Chilopsis linearis* (smaller in width, not length), *Olneya tesota* and *Larrea tridentata*), the entire leaf was used because it was nearly impossible to cut the lamina in half from the midvein. Thus, these species may have a slightly overestimated force-to-tear value since their midvein was included. The mass in kilograms (kg) recorded by the scale was then converted to a force and divided by the width of the sample to give force-to-tear (N mm^−1^) (Pérez-Harguindeguy *et al.* 2013). All species were clamped in a perpendicular direction except for *Larrea tridentata*, which was clamped parallel to its main axis due to strong directionality of the parallel midveins **[see ****Supporting Information—Fig. S5****]**.

### Leaf venation

Additional leaves from the same individuals were collected for venation analysis **[see ****Supporting Information—Appendix S5, Data S4****]**. For species with large leaves, segments of approximately 1 × 1 cm were cut, avoiding major veins; for species with smaller leaves, the entire leaf or several leaflets were used. Samples were placed in labelled tissue cassettes. A subset of large leaves were chemically processed whole, within a labeled mesh envelope, for whole-leaf imaging. All samples underwent the same chemical processing described below.

Samples were placed in a 5 % sodium hydroxide (NaOH) and water bath at 50 °C until partially transparent. Samples were then rinsed in a water bath to stop reactions and to wash away loose tissue. Samples were then placed in a 2.5 % sodium hypochlorite and water bath in order to bleach the leaf for staining. Once bleached, samples were then rinsed again in a water bath to remove any excess solution. Samples were then transferred to a 50 % ethanol and water solution for 1–2 minutes to prepare for staining. Samples were then placed in a 0.1 % safranin and 100 % ethanol solution for 30–60 minutes to stain. Afterward, the samples were stored in a 100 % ethanol solution until mounted on microscope slides. Samples ready for mounting were transferred to a 50 % ethanol/toluene or xylene solution for 3–5 minutes, then transferred to a 100 % toluene or 100 % xylene solution for another 3–5 minutes. Sample preservation changed between toluene and xylene due to chemical availability; both solutions work well for these procedures. Lastly, cassette and small leaf samples were mounted between a microscope slide and coverslip, while whole leaves were mounted between clear acetate sheets. Both sample types were mounted with xylene-based mounting medium.

**Figure 2.**
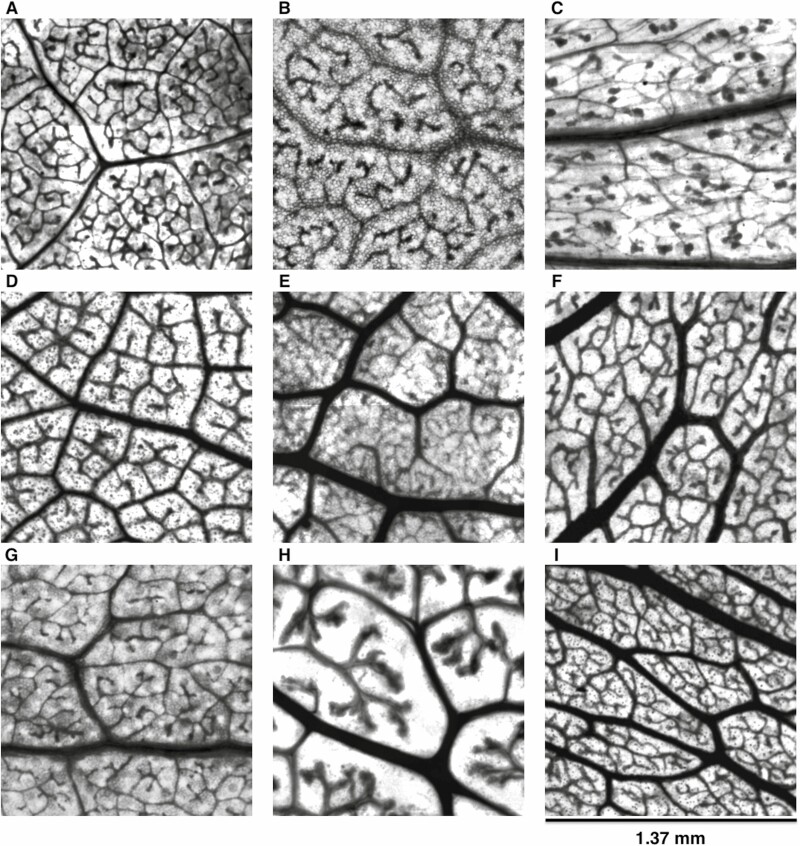
Leaf vein architecture obtained from desert leaf samples. Plant species: **A**) *Encelia farinosa*; **B**) *Rhus ovata*; **C**) *Larrea tridentata*; **D**) *Vauquelinia californica*; **E**) *Populus fremontii*; **F**) *Prosopis velutina*; **G**) *Chilopsis linearis*; **H**) *Simmondsia chinensis*; **I**) *Olneya tesota*.

**Figure 3.**
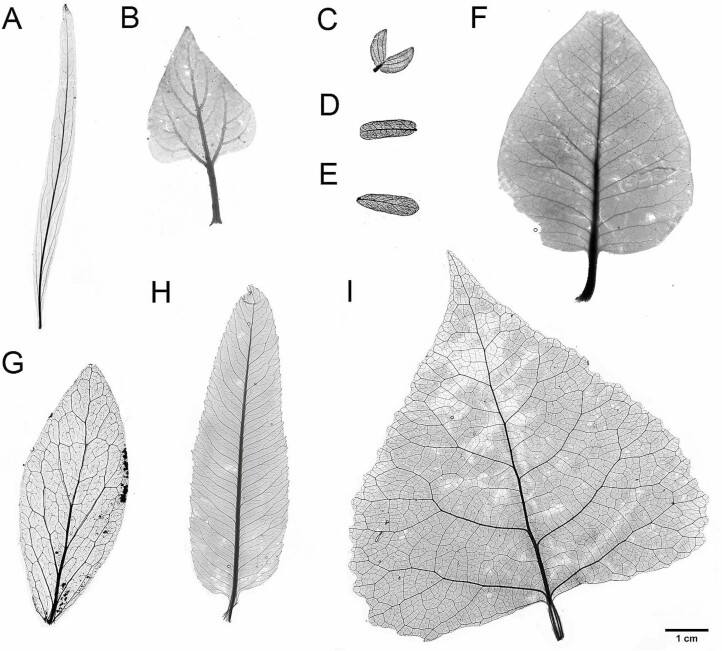
True-size vein architecture of select whole-leaf samples processed and analyzed in the present study. **A**) *Chilopsis linearis*, **B**) *Encelia farinosa*, **C**) *Larrea tridentata*, **D**) *Prosopis velutina*, **E**) *Olneya tesota*, **F**) *Rhus ovata*, **G**) *Simmondisia chinensis*, **H**) *Vauquelinia califronica*, **I**) *Populus fremontii*. *Larrea tridenta* shown as a full leaf, with leaflets attached to the petiole.

Once mounted and dried for at least a full day, the microscope slides were taken to Arizona State University’s Keck Lab. Microscopic images of the processed samples for vein network parameters were imaged using an inverted microscope (Evos FL Auto Live Cell Imaging System and Nikon Eclipse TE300 microscope; ThermoFisher, Waltham, MA, USA). Images were taken at 20× resolution, which resulted in images with a resolution of approximately 512 pixels mm^−1^ (Fig. 2). Separately, mounted whole leaves were placed on a light mat and then imaged using a robotic camera system that took image tiles of the samples following a tracked sequence. The image tiles were then stitched using the Image Composite Editor software (Microsoft Research, Redmond, WA, USA), which resulted in images with a resolution of approximately 145 pixels mm^−1^ (Fig. 3).

### Vein image processing

Microscope slide images were traced by hand in GIMP (version 2.10) within a representative sub-region of ~700 × 700 pixels **[see ****Supporting Information—Fig. S6****]** that does not include the midvein. Traced images were then processed via script (https://github.com/bblonder/venation_programs) in MATLAB in order to estimate minor vein density (i.e. length of veins per unit leaf area; mm^−1^) and minimum spanning tree (MST) ratio (values near 0 indicating highly reticulate networks and near 1 indicating highly non-reticulate networks; dimensionless, Xu *et al.*, 2021). Mean number of traced images per species was 7 ± 4 s.d.

Whole-leaf mount and whole-leaflet slide images were machine traced **[see ****Supporting Information—Fig. S6C****]** using LeafVeinCNN algorithm, developed by Xu *et al.* (2021). All parameters were set to defaults, except threshold values, which were selected manually to optimize segmentation results. Because the algorithm measures vein size, we truncated results to include only veins above a radius of 0.08 mm (i.e. value of ‘width_threshold’ in the program outputs), which are hereafter called ‘*major veins*’. The resulting images were used to calculate major vein density (mm^−1^) and MST ratio. The mean number of analyzed images per species was 2 ± 0.9 s.d.

### Phylogenetic data

A phylogenetic tree was generated for the species used in this study **[see ****Supporting Information—Fig. S7****]** using the V.PhyloMaker R package, based on the ‘GBOTB’ backbone tree, and using ‘scenario 3’ to resolve polytomies (Jin and Qian 2019; Smith and Brown 2018; Zanne *et al.* 2014). The tree was used to account for relatedness among species in regression analyses.

### Statistical analysis

All data were processed in R (version 3.6.1). Variation within traits and physiological rates across species was analyzed using ANOVAs. Physiological rate differences between species was quantified through a Tukey honest significant difference (HSD) multi-comparison *post hoc* test.

#### Questions 1 and 2

: Gas exchange measurements (*An, gsw,* and *Et*) were averaged for each species since leaves measured for gas exchange were not the same ones used for leaf trait estimates. A generalized least squares model of the form *An*, *gsw*, and *Et* ~ Treatment was fit to species-treatment means, and using a Brownian correlation structure based on the generated phylogeny. The significance of the treatment effect across all species was then determined via one-way ANOVAs. The differences between physiological rates (*An*, *Et*, *gsw*) and response to damage (*ΔAn* %, *ΔEt%*, *Δgsw%*) within species were assessed through paired *t*-tests (α = 0.05) on the pooled and per species data.

To test Hypothesis 1a/1b, we calculated Pearson correlations for each of LDMC, LMA, leaf area and force-to-tear against each of minor/major vein density and minimum spanning tree ratio also based on the generated phylogeny.

#### Question 3:

The percent change of the damage-induced *An*/*Et*/ *gsw* was calculated as


$$\begin{array}{l} \Delta An\%,\;\Delta gsw\%\;or\;\Delta Et\%=\\ \frac{\left(Treatment\;Mean\;per\;Species-Control\;Mean\;per\;Species\right)}{\left(Control\;Mean\;per\;Species\right)} \end{array}$$

A negative value means that treatment values were lower than control, while a positive value means that treatment values were greater than control.

We tested Hypothesis 2a/2b by determining the effect of each trait predictor on *ΔAn%*, *Δgsw%*, and *ΔEt%* via a set of generalized least squares models. Each generalized least squares regression model included a Brownian correlation structure based on the above phylogeny. Coefficient of determination (Cox & Snell pseudo-R^2^; Nakagawa and Schielzeth, 2013) and *P*-values for each model were calculated. Multivariate models were not used as the relatively low number of species relative to predictor variables made it difficult to assess predictor covariance. Predictor traits included LDMC, force to tear, minor and major vein densities, and minor and major MST ratios.

## Results

### Question 1: Gas exchange response to vein damage

**Figure 4.**
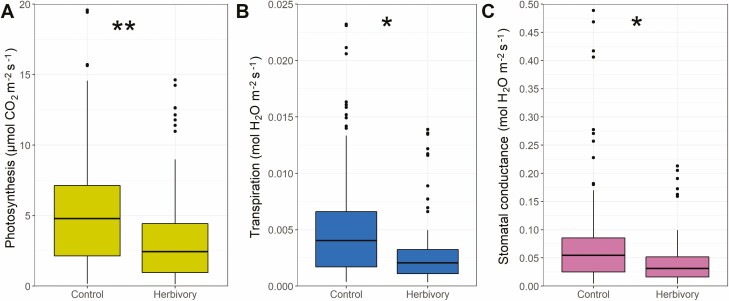
Gas exchange response to treatments (Control and Herbivory, i.e., simulated vein damage) aggregated across all species, for **A**) net photosynthesis; **B**) transpiration; C) stomatal conductance. *P*-values obtained through generalized least squares with phylogenetic correlation structure, where asterisks show the effect of treatments within each species (**P < 0.01, *P < 0.05).

For Question 1, we hypothesized that physiological responses would all be negatively affected by the simulated herbivory to the midvein. We found that average control net photosynthesis (*An*) was 5.32 ± 3.99 μmol CO_2_ m^−2^ s^−1^ (Fig. 4), and herbivory *An* 3.15 ± 3.18 μmol CO_2_ m^−2^ s^−1^ (−41 % difference, *P* < 0.01). Average transpiration (*Et*) was 5.11 ± 4.72 mmol H_2_O m^−2^ s^−1^ for control leaves, and 2.77 ± 2.63 mmol H_2_O m^−2^ s^−1^ for damaged leaves (−46 % difference, *P* < 0.05). Average stomatal conductance (*gsw*) was 0.074 ± 0.083 mol H_2_O m^−2^ s^−1^ for control leaves, and 0.039 ± 0.038 mol H_2_O m^−2^ s^−1^ for herbivory treatment (−47 % difference, *P* < 0.05).

Gas exchange rates varied widely among species for control and herbivory treatments **[see ****Supporting Information—Fig. S8****]**, with *Encelia farinosa* and *Larrea tridentata* having the highest and lowest rates (*P* < 0.001), respectively, for both treatments and across physiological responses. Although *E. farinosa* consistently had the highest rates among the species sampled, statistically, *L. tridentata* was consistently non-significantly different to *Populus fremontii* rates across all treatments and physiological rates **[see ****Supporting Information—Fig. S8****]**, although *L. tridentata* did have the lowest rates among the species measured. Interestingly, with exception of *E. farinosa*, stomatal conductance rates were non-significantly different across all the other species and both treatments **[see ****Supporting Information—Fig. S8C****]**. Mean maximum rates per treatment (all *Encelia farinosa*) were *An*= 9.61 ± 6.41 μmol CO_2_ m^−2^ s^−1^, *Et*= 14.13 ± 6.00 mmol H_2_O m^−2^ s^−1^ and *gsw*= 0.22 ± 0.15 mol H_2_O m^−2^ s^−1^ for *control*; and *An*= 6.28 ± 5.23 μmol CO_2_ m^−2^ s^−1^, *Et*= 8.41 ± 4.43 mmol H_2_O m^−2^ s^−1^ and *gsw*= 0.11 ± 0.07 mol H_2_O m^−2^ s^−1^ for *simulated herbivory*. While mean minimum rates (all *Larrea tridentata*) were *An*= 1.08 ± 0.74 μmol CO_2_ m^−2^ s^−1^, *Et*= 0.95 ± 0.62 mmol H_2_O m^−2^ s^−1^, and *gsw*= 0.01 ± 0.006 mol H_2_O m^−2^ s^−1^ for *control*; and *An*= 0.73 ± 0.39 μmol CO_2_ m^−2^ s^−1^, *Et*= 1.01 ± 0.39 mmol H_2_O m^−2^ s^−1^ and *gsw*= 0.12 ± 0.004 mol H_2_O m^−2^ s^−1^ for *simulated herbivory*.

**Figure 5.**
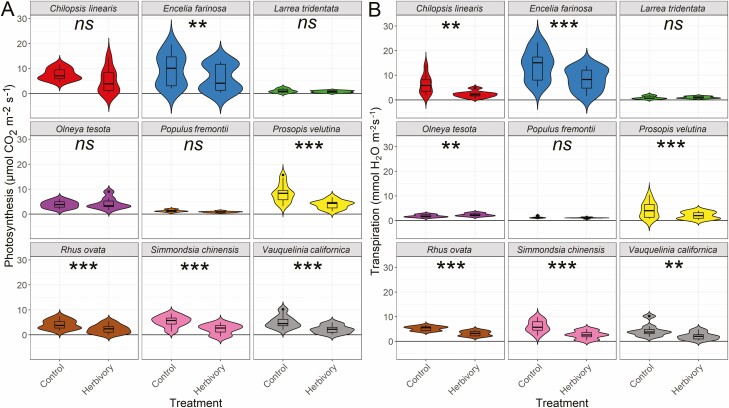
Violin plots of mean gas exchange response to treatments (Control and Herbivory, i.e. simulated vein damage) for each species, for photosynthesis (**A**) and transpiration (**B**). Asterisks refer to *P*-values showing the effect of treatments within each species (***P < 0.001, **P < 0.01, *P < 0.05, *ns* non-significant *P* > 0.05). Box plots within violins depicting median and confidence intervals. See **Supporting Information—Fig. S7** for stomatal conductance responses.

On average, *An*, *Et*, and *gsw* significantly declined (−37 ± 21 %, −28 ± 30 %, −27 ± 33 %, respectively; *P* < 0.001 for each) across species under simulated herbivory conditions (Fig. 5; **[see ****Supporting Information—Fig. S9****]**. Per species, average *ΔAn%* ranged between +10 and −55 %, with *Olneya tesota* (*ΔAn%=* +10 %, P = 0.39), *Larrea tridentata* (*ΔAn%*= −30 %, *P* = 0.17), and *Populus fremontii* (*ΔAn%*= −29 %, *P* = 0.44) not differing significantly from control leaves (Table 2; Fig. 5).

**Table 2.** Average percent change of physiological rates per species (*ΔAn*%, *ΔEt*%, *Δgsw*%), and respective significance values (*P*-value) obtained from paired *t*-test (α = 0.05) between control and herbivory mean physiological rates.

Species	*ΔAn%*	*P*-value	*ΔEt%*	*P*-value	*Δgsw%*	*P*-value
*Chilopsis linearis*	-30.9 ± 0.79%	<0.05	−46.3 ± 52.7%	<0.01	−40.9 ± 0.62%	<0.01
*Encelia farinosa*	-37.6 ± 0.04%	<0.01	−39.9 ± 0.10%	<0.001	−45.9 ± 19.0%	<0.001
*Larrea tridentata*	-29.9 ± 15.1%	0.17	13.3 ± 38.3%	0.35	16.7 ± 39.9%	0.17
*Olneya tesota*	10.2 ± 18.3%	0.39	27.5 ± 27.5%	<0.01	36.3 ± 30.4%	<0.01
*Populus fremontii*	−29.1 ± 23.6%	0.44	−16.1 ± 12.4%	0.67	−15.6 ± 16.6%	0.76
*Prosopis velutina*	−53.6 + 0.8%	<0.001	−51.5 ± 0.1%	<0.001	−50.8 ± 1.1%	<0.001
*Rhus ovate*	−52.7 ± 23.0%	<0.001	−40.0 ± 1.2%	<0.001	−39.3 ± 2.8%	<0.001
*Simmondsia chinensis*	−54.9 ± 35.3%	<0.001	−53.6 ± 24.9%	<0.001	−53.1 ± 26.9%	<0.001
*Vauquelinia californica*	−53.4 ± 12.1%	<0.001	−47.6 ± 15.5%	<0.01	−51.8 ± 17.9%	<0.01

Average *ΔEt%* ranged between +27 % and −54 % (Fig. 5), with *Larrea tridentata* and *Populus fremontii* having responses to damage matching those of control leaves (*ΔEt%=* +13 %, *P* = 0.79; −16 %, *P* = 0.67). Although the *Et* responses of *Olneya tesota* were different from control (*P* < 0.05), this was the only species that presented a substantial increase in *Et* after vein damage (*ΔEt%*= +27 %) (Fig. 5). Meanwhile, *Δgsw%* ranged between +36 and -53 % **[see ****Supporting Information—Fig. S9****]**. Similarly, to *ΔEt%*, *Larrea tridentata* and *Populus fremontii* responses to damage did not differ from control leaves (*Δgsw%*= +17 %, *P* = 0.57; −16 %, *P* = 0.76), and *Olneya tesota* had the most positive response to vein damage among the species studied (*Δgsw%* = +36 %; *P* < 0.01). Interestingly, these three species showed an increase in leaf water loss in comparison to the decline in *An. O. tesota* increased *An* after damage, but had lower rates of *Et* and *gsw*, although these were still an increase. Meanwhile *P. fremontii* still had reduced rates, but proportionally lost more *Et* and *gsw* than *An*; and *L. tridentata* had a significant decline in *An*, but also significantly increased *Et* and *gsw* (Table 2).

### Question 2: leaf trait tradeoffs

Leaf traits also varied widely across species. Mean LDMC was 0.47 ± 0.22 (sd) g/g (*P* < 0.001), mean force to tear was 0.88 ± 0.52 N mm^−1^ (*P* < 0.001), mean leaf minor vein density was 12.96 ± 2.57 mm^−1^ (*P* < 0.001), major vein density (0.97 ± 0.67 mm^−1^, *P* < 0.001), mean MST ratio for minor (0.78 ± 0.07, *P* < 0.001), and major (0.92 ± 0.10, *P* < 0.01) veins.

We hypothesized for Question 2 that vein density would be positively correlated to force to tear as high vein densities would require more force to tear the leaf apart (i.e. tougher to chew and tear). We found that force to tear was not correlated to vein densities (*P* = 0.59 (minor), *P* = 0.53 (major)) or MST ratios (*P* = 0.74 (minor), *P* = 0.73 (major)), indicating the absence of a tradeoff among these traits **[see ****Supporting Information—Table S1****]**. Interestingly, LMA was the only trait strongly correlated to any venation trait, minor vein density (*P* < 0.01) and marginally to minor MST ratio (*P* = 0.05). However, LMA was not correlated to *ΔAn%*, *ΔEt%* and *Δgsw%* (results not shown).

### Question 3: trait drivers of gas exchange response

**Figure 6.**
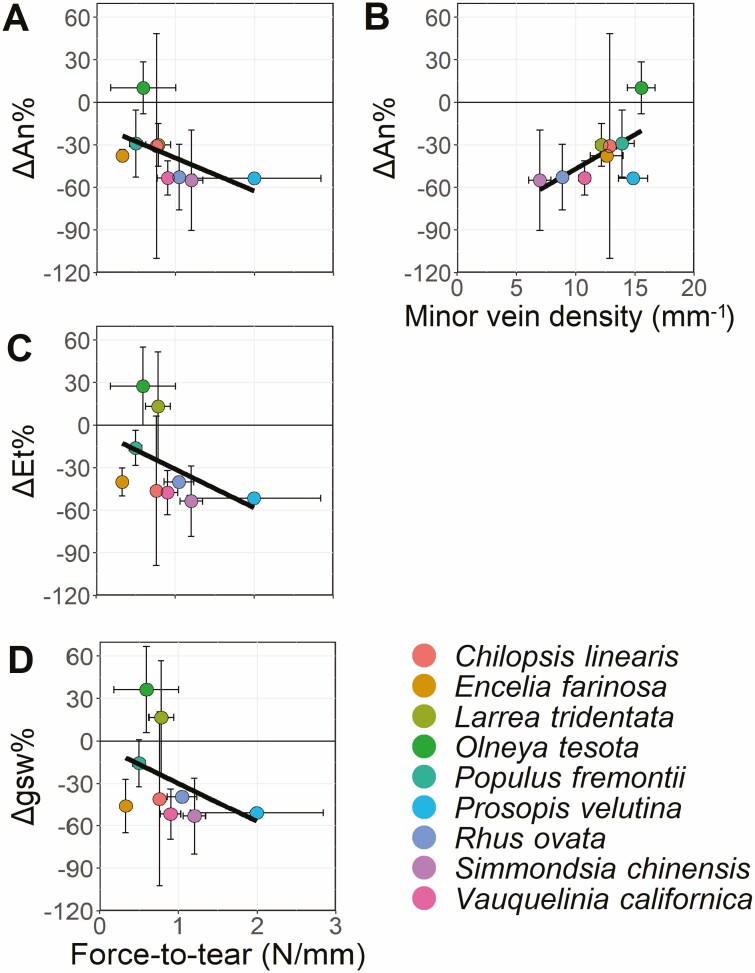
Significant linear relationships of mean responses to damage of photosynthesis (*ΔAn%*, **A**, **B**), transpiration (*ΔEt%*, **C**) stomatal conductance (*Δgsw%*, **D**) to force-to-tear and minor vein density. The remaining trait relationships were all non-significant. See **Supporting Information—Fig. S10** for full panel of linear regressions, and Table 3 for complete statistical results.

For Question 3, we hypothesized that venation traits would be the best mitigator against the effects of the simulated herbivory (less negative or positive *ΔAn%*, *ΔEt%* and *Δgsw%*), which would enable carbon assimilation and water flux to continue after damage. We found that *ΔAn%* was negatively associated with force to tear (*P* < 0.05), and positively with minor vein density (*P* = 0.05, Table 3; Fig. 6). These relationship trends were consistent across *ΔEt%* and *Δgsw%* (Table 3). Other relationships rarely met the *P* < 0.05 statistical thresholds or were marginally significant at *P* < 0.10 threshold, such as LDMC, major vein density, major and minor vein MST ratios (Table 3). *ΔAn%* was significantly influenced by force-to-tear and minor vein density, while *ΔEt%* and *Δgsw%* were only correlated to force to tear (*P* < 0.05 and *P* = 0.06, respectively).

**Table 3.** Statistical results from the generalized least squares correlation between leaf traits and physiological responses (*ΔAn%*, *ΔEt%*, *Δgsw%*) taking phylogeny into consideration. Parameters shown are *P*-value, pseudo-*R*^2^ (Cox and Snell), minimum (min), and maximum (max) value ranges of the averages. Dark grey values represent statistically significant relationships (α = 0.05), light grey values represent marginally significant relationships (α = 0.10).

Leaf Trait	*ΔAn%*			ΔEt%			Δgsw%			Range	
	*R* ^*2*^	*P*-value	Slope	*R* ^*2*^	*P*-value	Slope	*R* ^2^	*P*-value	Slope	Min	Max
Force-to-tear (N/mm)	0.48	0.02	−0.30	0.38	0.04	−0.37	0.34	0.06	−0.38	0.33	1.99
LDMC (g/g)	0.13	0.26	-0.78	0.0001	0.97	−0.03	7.5 × 10^−6^	0.99	−0.01	0.29	0.59
Minor vein density (mm^−1^)	0.33	0.06	0.05	0.20	0.16	0.06	0.20	0.16	0.06	8.89	56.79
Major vein density (mm^−1^)	0.04	0.52	−0.09	0.23	0.13	−0.28	0.18	0.19	−0.27	0.21	1.77
Minor vein MST ratio	0.04	0.55	−0.54	0.03	0.63	−0.61	0.01	0.72	−0.49	0.69	0.95
Major vein MST ratio	0.08	0.38	0.71	0.19	0.17	1.49	0.18	0.17	1.64	0.76	1.00

Slopes of major vein effects were opposite to minor vein effects, with the flux response to damage increasing with the increase of major veins, but decreasing with the increase of minor veins. The opposite was observed for major and minor vein MST ratios, where leaves with higher major vein reticulation patterns (low MST ratio) were the most affected by the midvein damage. Minor vein MST ratios showed opposite trends to major vein MST ratios, but the slopes were nearly constant across species reticulation and branching patterns. Considering that these relationships were non-significant (Table 3), especially due to within species variation, these trends might not hold with larger sample sizes.

## Discussion

We found that (Question 1) experimental damage to midveins caused diverse impacts on gas exchange that were usually but not always negative; (2) there was no tradeoff between mechanical and venation traits, tentatively supporting Hypothesis 1b; and (3) force to tear and possibly minor vein density increased damage resilience, weakly supporting Hypothesis 2a.

### Question 1: gas exchange response to vein damage

Our results contribute empirical data for desert plants to a growing number of studies that have examined the consequences of experimental damage on leaf gas exchange (Baldwin 1990; Trumble *et al.* 1993; Visakorpi *et al.* 2018; Welter 2019). Many studies have shown that gas exchange rates transiently increase after damage, in order to compensate for reduced leaf area (Trumble *et al.* 1993; Welter 2019). Other studies instead have shown no effects on gas exchange, (Aldea *et al.* 2005) or negative effects (Warrington *et al.*, 1989; Tang *et al.* 2006; Delaney *et al.* 2008; Visakorpi *et al.* 2018). Some of the variation is likely due to differences in the species studied and the type of damage considered (e.g. cutting of lamina, severing of midvein, leaf-mining).

Our study now shows that in desert plants exposed to midrib damage, the effects on gas exchange are primarily negative, with only one species showing, on average, positive responses. This finding is likely due to the interaction between the severity of the damage (loss of most of the primary water-conducting pathway) and the extremity of the environment (very high atmospheric water demand during a hot summer). In some cases, post-wounding gas exchange could be a transient effect (Marchin *et al.* 2010) and/or modulated by hormonal signals (de Dios *et al.*, 2021; Lin *et al.*, 2021) but these response times vary widely across species (Brodribb *et al.*, 2016). This can explain the response observed in the field from *Prosopis velutina* and *Chilopsis linearis* leaves, in which leaves showed signs of desiccation and severe wilting, respectively, in less than 48 hours after pilot wounding as it is not regulated by stomata, but rather, leaves via the wounds. Due to these rapid responses to damage, gas exchange measurements had to be made between 12 and 24 hours after experimental treatment.

Alternatively, the magnitude of the response to vein wounding could be related to the species’ water use strategy and leaf economics. For example, some Sonoran desert species maintain a high LMA, a constant photosynthetic activity, high leaf conductance, and a less strict regulation of leaf water potentials (i.e. anisohydric strategy; González-Rebeles *et al.*, 2021). Conversely, other species adopt a more conservative strategy by maintaining a low stomatal conductance, high minimum leaf water potentials, and possibly more leaf shedding to prevent xylem cavitation (i.e. isohydric strategy; González-Rebeles *et al.*, 2021). Therefore, we can assume that isohydric species would be the most affected if xylem damage occurs from vein wounding. Taking this previous study into consideration, we analyzed the physiological response data against the water use strategies of species analyzed in the present study based on the data shown in Aparecido *et al.* (2020). We found that there is a marginally significant relationship between responses to herbivory and water use strategy in terms of transpiration and stomatal conductance, although no relation was detected with net photosynthesis (**Fig. S11**). While the expression of isohydric behaviour was only marginally linked to greater vein damage, these results indicate that water use strategy could still be considered as a potential predictor of a species response to vein damage. However, we caution as water use strategy can be highly plastic across seasons, and arid adapted species could switch seasonally from isohydric to anisohydric behaviour (Guo *et al.*, 2020, 2022).

Overall, we found significant variation in species’ damage resilience, which likely has very different outcomes for long-term carbon gain (Kikuzawa and Lechowicz 2006). In some species, photosynthesis was depressed by nearly 100 % during the measurement period. The long-term consequences of this depression depend on whether recovery of photosynthesis and transpiration is possible, and on whether damage drives early leaf senescence (Lin *et al.*, 2021). We were not able to measure either effect, but we do note that some species (*Prosopis velutina*, *Chilopsis linearis*) dropped damaged leaves within a week of experimental treatment.

### Question 2: predictors of herbivory resistance

We found no evidence for tradeoffs (or synergies) among venation and mechanical traits, suggesting that these two sets of traits represent independent axes of variation in desert species (Hypothesis 1b). This finding is consistent with a previously suggested existence of selection for high leaf lifespan relative to hydraulic performance in desert plants (de Boer *et al.* 2016). Under this perspective, the absence of a tradeoff may suggest that the venation network represents a relatively small construction cost relative to the rest of a leaf in this biome. Such differentiation of trait couplings and allocation tradeoffs by biome could potentially explain why our finding here is apparently contrary to the mechanical—venation relationships found in a prior study of tropical trees (Blonder *et al.* 2018). However, this perspective is difficult to reconcile with the diversity of leaf forms included in this study, including several non-sclerophyllous species (e.g. the riparian-associated *Populus fremontii*).

### Question 3: predictors of herbivory resilience

Our results provide evidence that force to tear, in addition to minor vein architecture (i.e. force-to-tear), may provide resilience of gas exchange to experimental damage (Hypothesis 2a), especially for photosynthesis. These findings build on a prior experimental study (Sack *et al.* 2008), but are differentiated here by a focus on gas exchange (rather than hydraulic conductance), and consideration of both major and minor vein traits (rather than palmate vs pinnate major venation), and inclusion of desert species (instead of temperate trees). The findings of our study and of Sack (2008) are in broad conceptual agreement, i.e. that more flow pathways can mitigate the effects of damage. However we did not find support for higher major vein density either positively or negatively influencing resilience, suggesting that the minor vein pathways are actually more relevant in these species. Additionally, we surprisingly did not find support for network reticulation either positively or negatively influencing resilience. This latter finding runs contrary to predictions of theory for network reticulation (Katifori *et al.* 2010) where looping should allow for more redistribution in cases of obstacles such as herbivory (Katifori *et al.* 2010) or hydraulic failure (Brodribb *et al.* 2016). The results also do not support the perspective that venation networks with greater sectoriality (less reticulation) should be better able to isolate damage, e.g. propagating embolisms or introduced infectious diseases, in turn reducing the negative impacts of isolated damage events (Hypothesis 2b). Alternatively, selection for resilience may be more important than selection for sectoriality in these desert species—a pattern worth exploring in other taxa with divergent architectures, e.g. *Gingko*, as well as many ferns with limited or no reticulation (Price and Weitz, 2014). The low number of species we were able to consider, plus the limitations of the simulated herbivory experiments used, may have increased the likelihood of obtaining a null result. Future work with fewer methodological limitations may provide a more conclusive experimental test of these hypotheses.

We are also unsure why certain species’ photosynthetic rates declined after damage but increased transpiration. Among the species observed that showed this trend, not all had the wound included in the cuvette chamber, thus excluding the possibility that the excess water loss was due to an open vein wound not yet stable post-damage (Rasulov *et al.*, 2019). Time of day also did not influence this pattern, excluding the possibility that transpiration increased to provide cooling during hot conditions (Lin *et al.*, 2017). It remains unclear if this response is functional or not.

### Other limitations

The mechanical simulation of herbivory employed here may be an incomplete proxy for insect herbivory. Differences may arise due to the absence of chemical and mechanical cues sensed by plants, which in turn may impact the induction of plant signaling and defense pathways (Wu and Baldwin 2010; Waterman *et al.* 2019). Post-damage emission of volatiles (Rasulov *et al.* 2019), changes in gene regulation (Arimura *et al.* 2000) and signalling (Havko *et al.* 2020) are known to drive complex ecophysiological responses. We were not able to assess these effects due to the scope of this study.

This study was framed primarily in terms of herbivory defense. However, the effect of herbivory is best understood not only in terms of the outcomes of defense, given that herbivory has occurred, but also in terms of the probability of herbivory occurring. We were not able to assess this key second component in this study, i.e. both the natural frequency of herbivory in these desert taxa, and the type of damage incurred (chewing/cutting, mining, vein severing, complete leaf removal, etc.). We expect that all forms variously occur, but do not know their relative prevalence.

Reticulation is thought to be a key defense for latex-producing species defending against vein-piercing insects (Dussourd and Eisner 1987). We were not able to directly assess the role of laticifer architecture in this study, as none were latex-producing, and exudate/resin production in these species was weak. With few exceptions (e.g. milkweeds, dogbanes) latex production is rare in this desert flora, suggesting that tests of this hypothesis would be better explored in other contexts.

Although caution was taken when damaging leaf midveins and measuring gas exchange, it is worth noting that the current study had possible shortcomings that affected the results. Firstly, due to the small size of some leaflets (e.g. *Prosopis velutina*, *Larrea tridentata*, *Olneya tesota*), it is uncertain if the puncture went through the midvein. Thus, it is possible that insignificant gas exchange results might have been due to failure in puncturing the midvein completely or partially. Furthermore, even though plants were measured in the morning (8 AM–1 PM), leaves that were measured closer to midday (11 AM–1 PM) might have underestimated absolute values of gas exchange for *both treatments* in comparison to leaves measured before 11 AM **[see ****Supporting Information—Table S2****]**. Since the study was conducted late June in Phoenix, AZ, it is possible that midday gas exchange measurements for both control and treatment leaves were reduced in response to heat stress (i.e. air temperature around 40 °C, Aparecido *et al.* 2020).

However, the key limitation of this study was the relatively small sample size that were required to be measured in the field for only two days due equipment availability. Limited replication of measurements and treatments meant it was difficult to distinguish between measured patterns arising from sampling variation or from real biological processes.

## Conclusion

Responses to experimental damage were variable among nine Sonoran Desert species. Force to tear was a key predictor of responses, promoting leaf resilience to experimental damage; while leaf minor vein density was marginally important, in contrast to major vein density or minor/major vein MST ratio. Though more experimental and statistical evidence will ultimately be required to fully assess the hypotheses, venation network architecture variation may influence plant defense in ways differing from more widely-considered structural and chemical defenses.

## Supporting Information

The following additional information is available in the online version of this article –

**Figure S1.** A) Example of how herbivory was simulated in simple leaf samples. B) Demonstration of how herbivory was simulated in compound leaf species.

**Figure S2.** Examples of *in situ* leaf damage in native Sonoran desert species. A) Arizona ash leaves (*Fraxinus velutina*) carved by leaf-cutter bees (*Megachile* sp.) for hive building (Phoenix, AZ, USA); B) Fremont cottonwood (*Populus fremontii*) housing *Coptodisca* sp. larvae that consume leaf tissue and veins, near Yuma, AZ, USA; C) Fremont cottonwood leaves consumed by winter moth caterpillars (*Operophtera brumato*; Page, AZ, USA).Photo sources: Luiza Aparecido.

**Figure S3.** A and B—Gas exchange measurements taken on a desert rosewood (*Vauquelinia californica*).

**Figure S4.** Example of an ideal tearing of a leaf sample with the tensometer, where the sample is torn about halfway.

**Figure S5.** An example of two leaves with different venation structures, (a) being parallel veins and (b) being perpendicular veins with more branching. The black solid lines represent the portions cut out of the leaf if it was too large to fit the 3.8 x 0.6 cm maximum parameter. The red dashed lines represent where the ideal tearing would happen, approximately in the middle of the parameter/cut-out and between secondary veins. Therefore, leaf (a) would be clamped from the left and right end of the cut-out, whereas leaf (b) would be clamped from the top and bottom. Since creosote (*L. tridentata*) was too small to be cut, the entire leaflet was used, but the sample was placed according to the guidelines based on vein orientation above. Figure adapted from ‘Fig. 2’ of the online teaching resource ‘Biology Majors II’, found here: https://courses.lumenlearning.com/wm-biology2/chapter/leaves/.

**Figure S6.** A) A venation image example from a section of a leaf microscopic sample of *Chilopsis linearis* prior to being traced. B) The same sample once the vein structure had been traced. C) A separate *Chilopsis linearis* whole-leaf sample after being artificially traced by the LeafVeinCNN machine learning algorithm. Panel A and B are 1.37 mm x 1.37 mm, while C is approximately 6.00 cm x 3.50 cm.

**Figure S7.** Inferred phylogenetic tree illustrating evolutionary relationships among the focal species.

**Figure S8.** Physiological rates (A: *An*, net photosynthesis (μmol CO_2_ m^−2^ s^−1^); B: *Et*, transpiration (mmol H_2_O m^−2^ s^−1^); C: *gsw*, stomatal conductance (mol H_2_O m^−2^ s^−1^)) *between* species per treatment (control and simulated herbivory). Analysis done on leaf-level dataset, instead of plant-level. Letters represent degrees of significance (α=0.05) based on Tukey’s HSD test. Variation of physiological rates *across* species per treatment were highly significant (P<0.001) for all.

**Figure S9.** Violin plots of mean gas exchange response to treatments (Control and Herbivory, i.e., simulated vein damage) for each species, for stomatal conductance. Asterisks refer to P-values showing the effect of treatments within each species (‘***’ P<0.001, ‘**’ P<0.01, ‘*’ P<0.05, *ns* non-significant P>0.05). Violins contain boxplots showing median and confidence interval per species.

**Figure S10.** Complete panel of the linear relationships between mean photosynthesis (*ΔAn%*, A–E), transpiration (*ΔEt%*, F–J) and stomatal conductance (*Δgsw%,* K–O) response to damage and leaf traits (force-to-tear, leaf dry matter content (LDMC), minor and major vein densities, and minor and major vein MST ratios) across species. Black solid regression lines correspond to significant relationships (*P* < 0.10), and black dotted lines to non-significant relationships (*P* > 0.10). For complete statistical results, see Table 3.

**Figure S11.** Water use strategy (Aniso- and isohydry) relationships to physiological responses to simulated herbivory (From left to right: photosynthesis (ΔAn%), transpiration (ΔEt%) and stomatal conductance (Δgsw%)). Water use strategies were obtained from Aparecido *et al.* (2020, *Ecology Letters*). ‘*ns*’ represents non-significant relationships (*P* > 0.10) based on Analysis of Variance (ANOVA) analysis.

**Table S1.** Pearson correlation matrix of leaf traits studied. Bold and colored values are statistically significant relationships; light gray—*P* < 0.01, Gray—*P* < 0.05, Dark gray—P<0.10. Full trait names: ‘Minor.VD’- minor vein density, ‘Major.VD’—major vein density, ‘Minor.MSTRatio’—minor vein MST ratio, ‘Major.MSTRatio’-major vein MST ratio, ‘FT’—force-to-tear, ‘LMA’—leaf mass per area, ‘LDMC’—leaf dry matter content, ‘LA’—leaf area.

**Table S2.** Sequence of gas exchange measurements.

plad002_suppl_Supplementary_Material

plad002_suppl_Supplementary_Data_S1

plad002_suppl_Supplementary_Data_S2

plad002_suppl_Supplementary_Data_S3

plad002_suppl_Supplementary_Data_S4

plad002_suppl_Supplementary_Data_S5

## Data Availability

Data are available as supporting material through the AoB Plants online system.
