# Supplementary material for: Vein network redundancy and mechanical resistance mitigate gas exchange losses under simulated herbivory in desert plants
Source: AoB Plants. 2023 Jan 24;15(2):plad002. doi: 10.1093/aobpla/plad002 (PMC10029807; doi:10.1093/aobpla/plad002)
Supplement: plad002_suppl_Supplementary_Material [file plad002_suppl_supplementary_material.pdf]

Duarte et al. - SUPPORTING INFORMATION

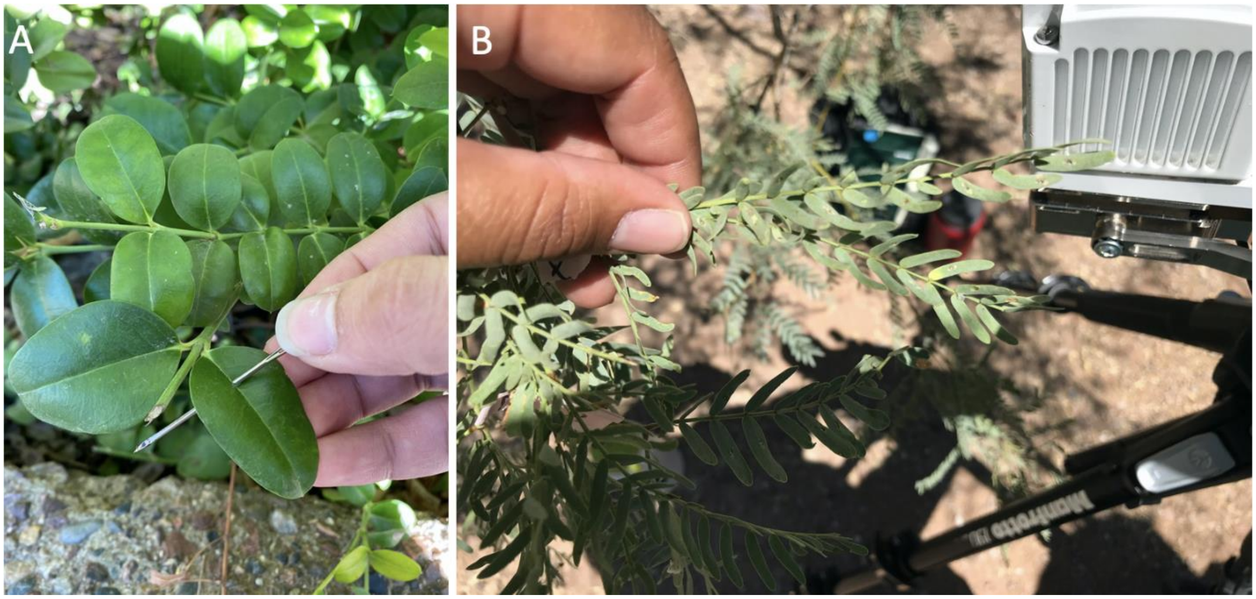

Figure S1. A) Example of how herbivory was simulated in simple leaf samples. B) Demonstration of how herbivory was simulated in compound leaf species.

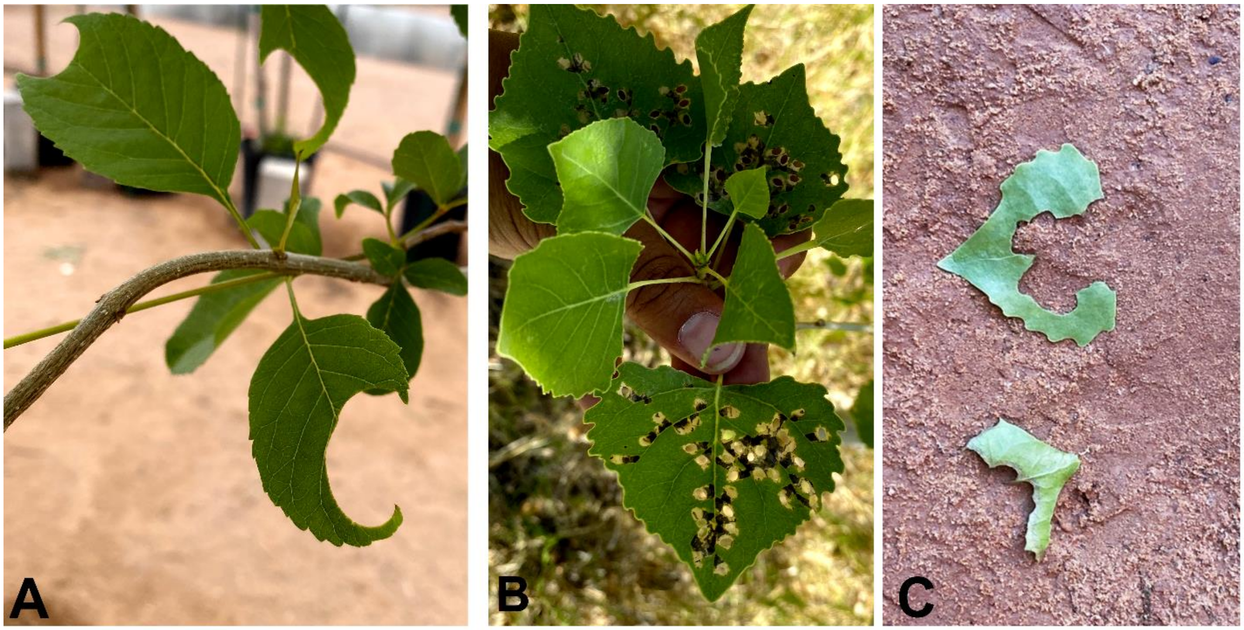

Figure S2. Examples of *in situ* leaf damage in native Sonoran desert species. A) Arizona ash leaves (*Fraxinus velutina*) carved by leaf-cutter bees (*Megachile* sp.) for hive building (Phoenix, AZ, USA); B) Fremont cottonwood (*Populus fremontii*) housing *Coptodisca* sp. larvae that consume leaf tissue and veins, near Yuma, AZ, USA; C) Fremont cottonwood leaves consumed by winter moth caterpillars (*Operophtera brumato*; Page, AZ, USA).Photo sources: Luiza Aparecido.

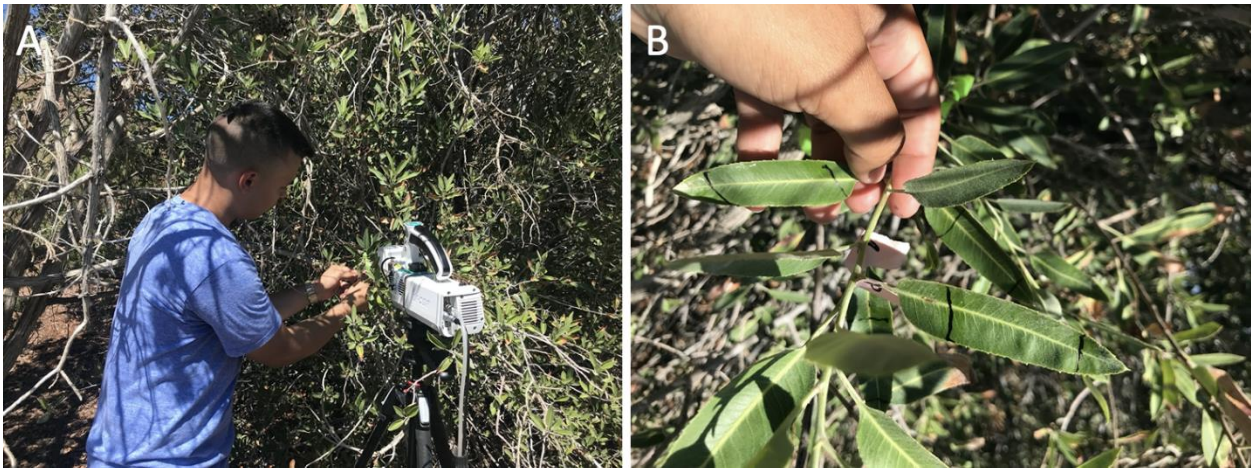

Figure S3. A and B - Gas exchange measurements taken on a desert rosewood (*Vauquelinia californica*).

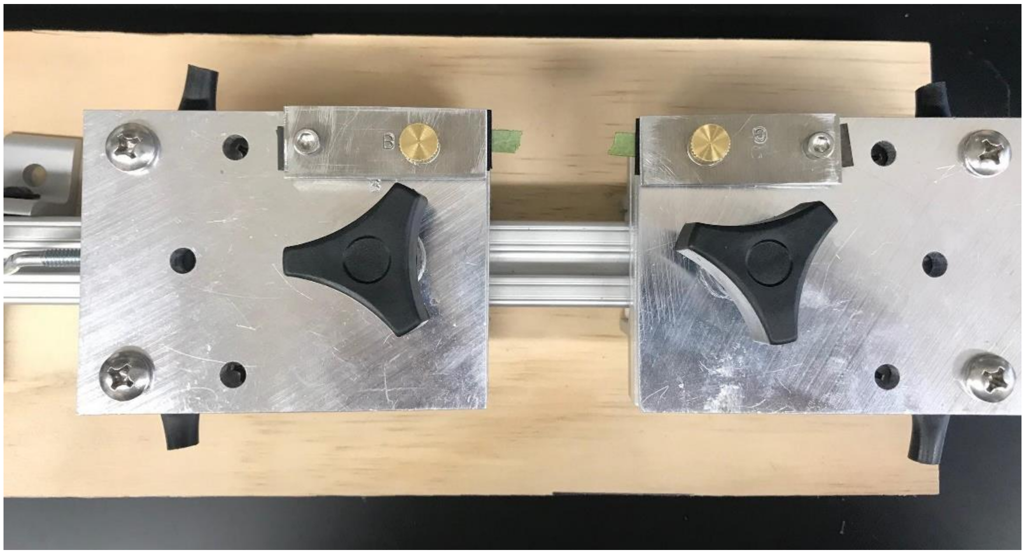

Figure S4. Example of an ideal tearing of a leaf sample with the tensometer, where the sample is torn about halfway.

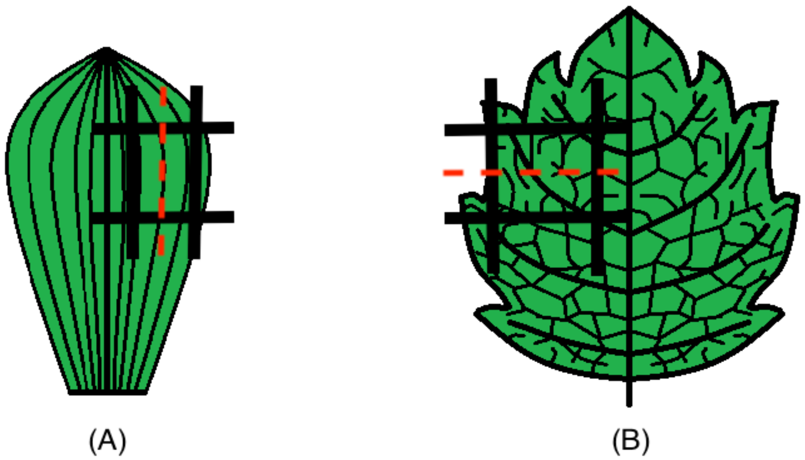

Figure S5. An example of two leaves with different venation structures, (a) being parallel veins and (b) being perpendicular veins with more branching. The black solid lines represent the portions cut out of the leaf if it was too large to fit the 3.8 x 0.6 cm maximum parameter. The red dashed lines represent where the ideal tearing would happen, approximately in the middle of the parameter/cut-out and between secondary veins. Therefore, leaf (a) would be clamped from the left and right end of the cut-out, whereas leaf (b) would be clamped from the top and bottom. Since creosote (*L. tridentata*) was too small to be cut, the entire leaflet was used, but the sample was placed according to the guidelines based on vein orientation above. Figure adapted from "Figure 2" of the online teaching resource "Biology Majors II", found here: https://courses.lumenlearning.com/wm-biology2/chapter/leaves/.

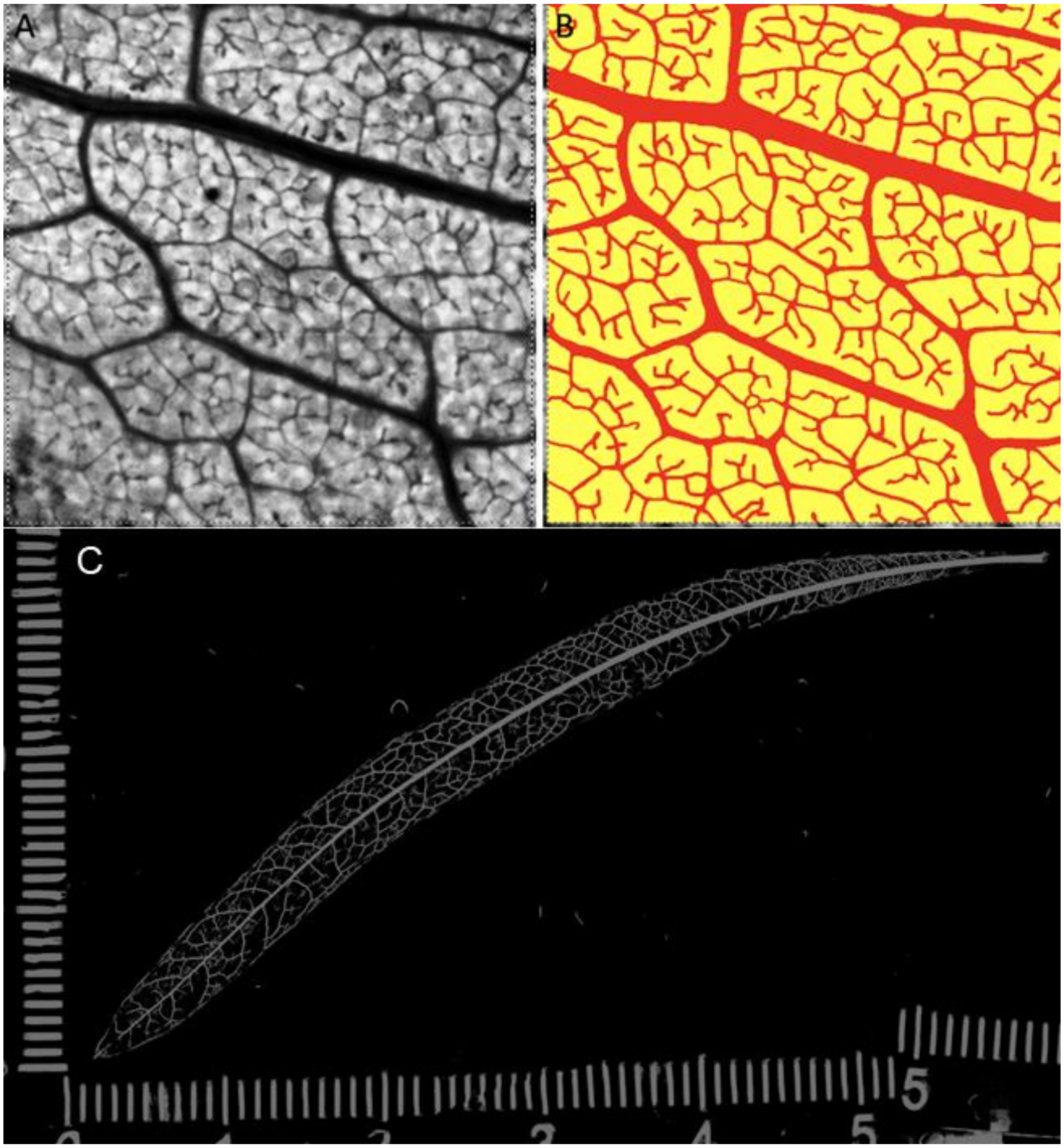

Figure S6. A) A venation image example from a section of a leaf microscopic sample of *Chilopsis linearis* prior to being traced. B) The same sample once the vein structure had been traced. C) A separate *Chilopsis linearis* whole-leaf sample after being artificially traced by the LeafVeinCNN machine learning algorithm. Panel A and B are 1.37 mm x 1.37 mm, while C is approximately 6.00 cm x 3.50 cm.

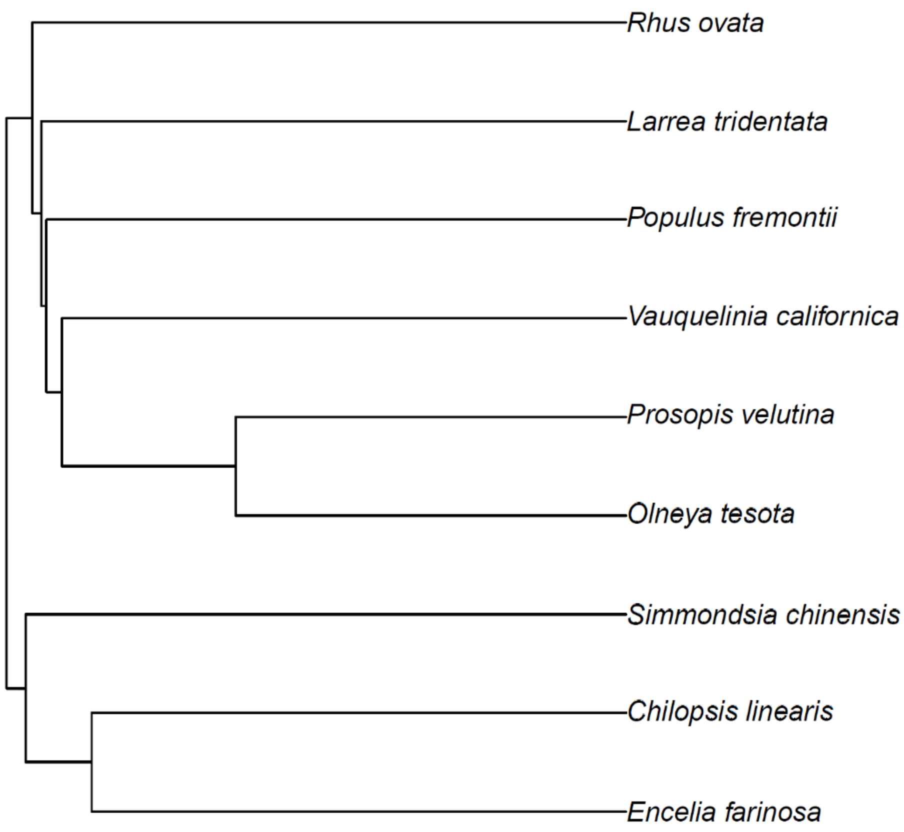

Figure S7. Inferred phylogenetic tree illustrating evolutionary relationships among the focal species.

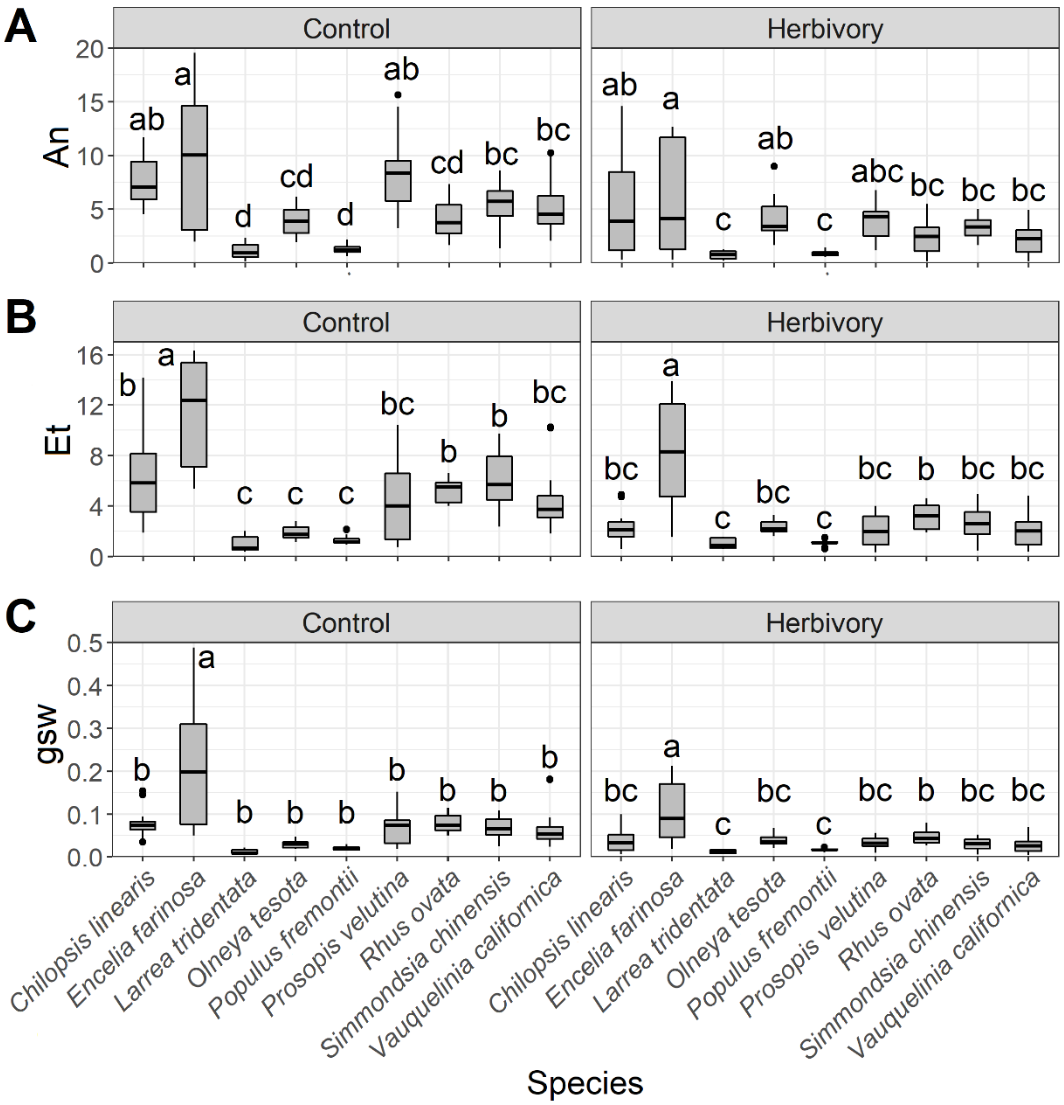

Figure S8. Physiological rates (A: *An*, net photosynthesis (µmol CO_2 m^{-2} s^{-1}); B: *Et*, transpiration (mmol H_2O m^{-2} s^{-1}); C: *gsw*, stomatal conductance (mol H_2O m^{-2} s^{-1})) *between* species per treatment (control and simulated herbivory). Analysis done on leaf-level dataset, instead of plant-level. Letters represent degrees of significance (α=0.05) based on Tukey's HSD test. Variation of physiological rates *across* species per treatment were highly significant ($P<0.001$) for all.

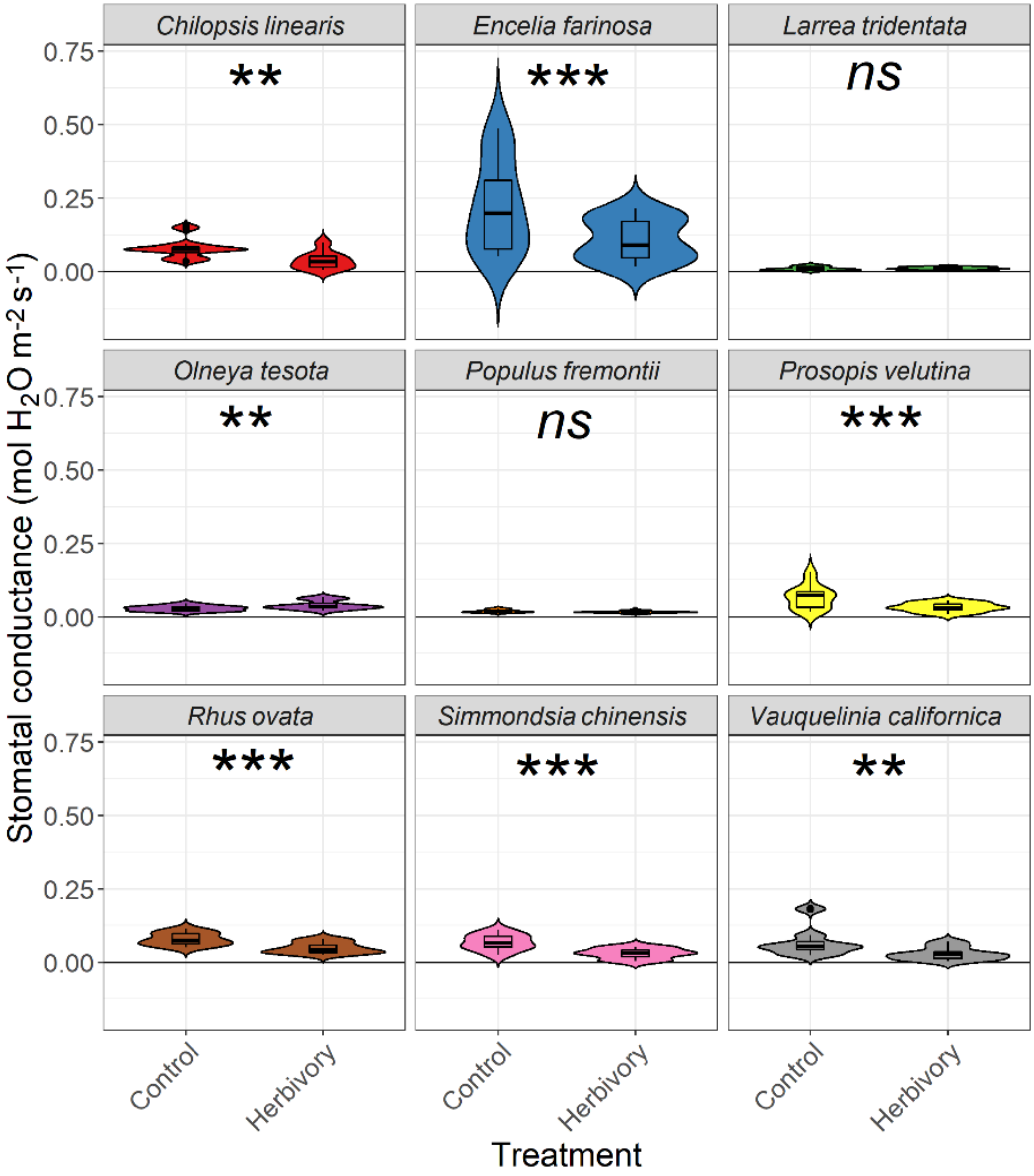

Figure S9. Violin plots of mean gas exchange response to treatments (Control and Herbivory, i.e., simulated vein damage) for each species, for stomatal conductance. Asterisks refer to P-values showing the effect of treatments within each species (‘***’ P<0.001, ‘**’ P<0.01, ‘*’ P<0.05, *ns* non-significant P>0.05). Violins contain boxplots showing median and confidence interval per species.

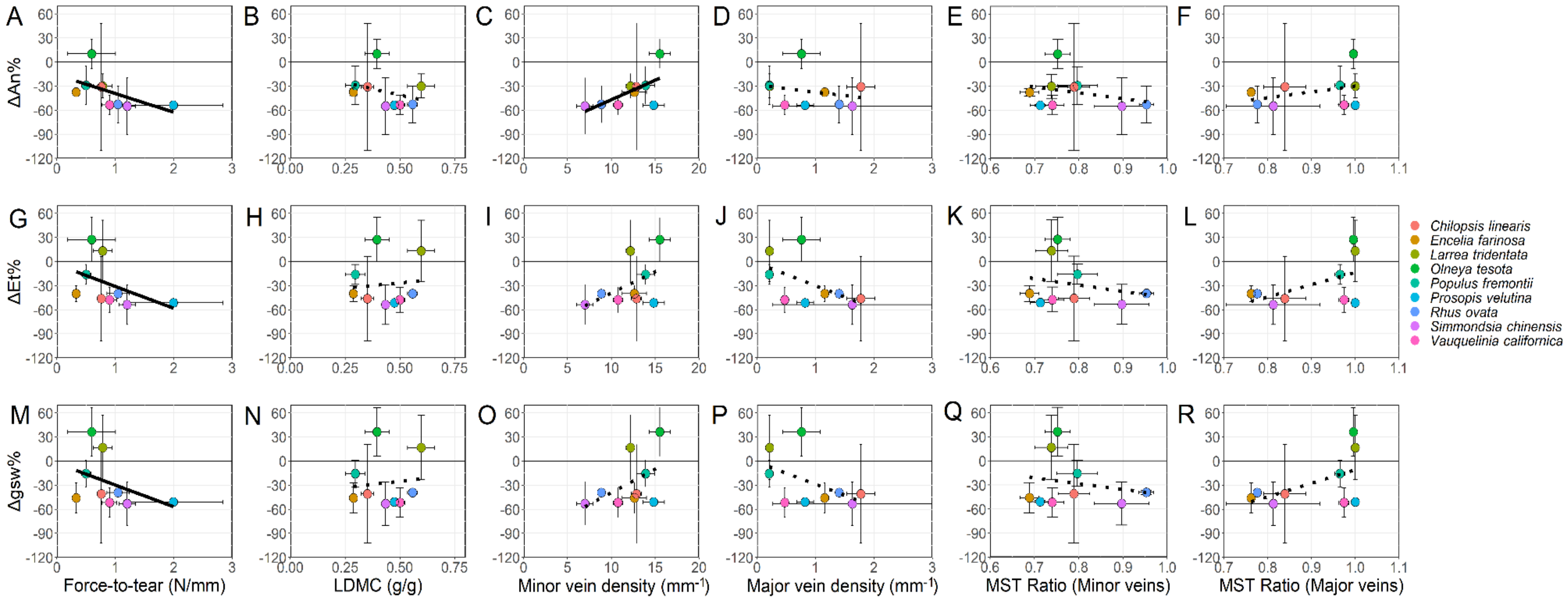

Figure S10. Complete panel of the linear relationships between mean photosynthesis (*ΔAn%*, A-E), transpiration (*ΔEt%*, F-J) and stomatal conductance (*Δgsw%,* K-O) response to damage and leaf traits (force-to-tear, leaf dry matter content (LDMC), minor and major vein densities, and minor and major vein MST ratios) across species. Black solid regression lines correspond to significant relationships ($P<0.10$), and black dotted lines to non-significant relationships ($P>0.10$). For complete statistical results, see Table 3.

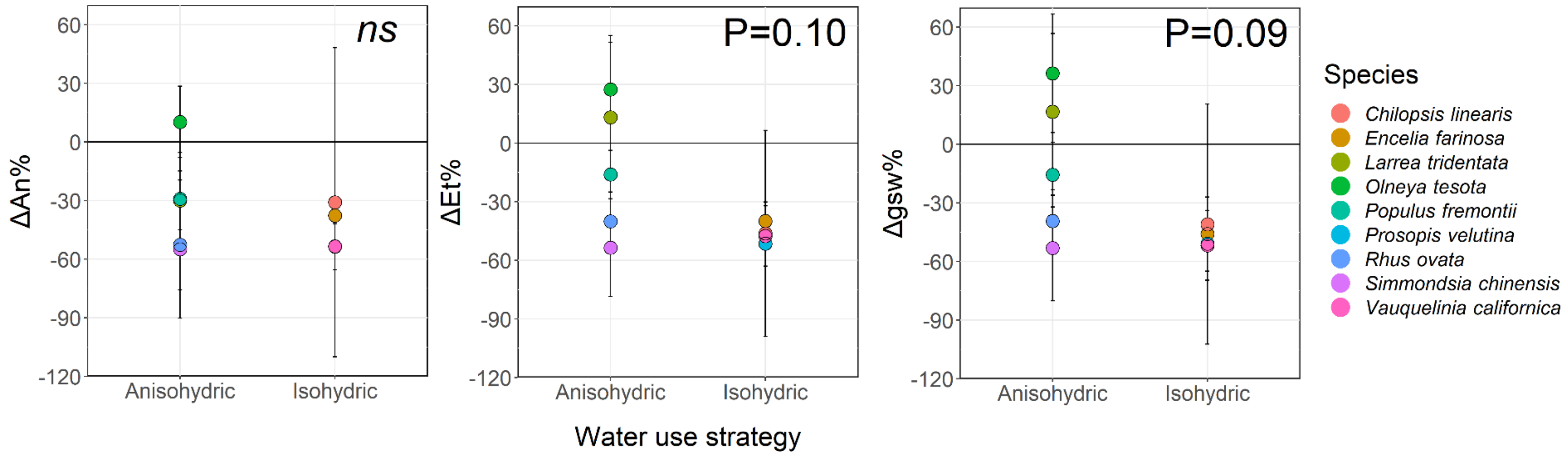

Figure S11. Water use strategy (Aniso- and isohydry) relationships to physiological responses to simulated herbivory (From left to right: photosynthesis (ΔAn%), transpiration (ΔEt%) and stomatal conductance (Δgsw%)). Water use strategies were obtained from Aparecido et al. (2020, *Ecology Letters*). '*ns*' represents non-significant relationships (P>0.10) based on Analysis of Variance (ANOVA) analysis.

Table S1. Pearson correlation matrix of leaf traits studied. Bold and colored values are statistically significant relationships; Light gray – P<0.01, Gray – P<0.05, Dark gray – P<0.10. Full trait names: 'Minor.VD'- minor vein density, 'Major.VD'- major vein density, 'Minor.MSTRatio'- minor vein MST ratio, 'Major.MSTRatio'-major vein MST ratio, 'FT'- force-to-tear, 'LMA' – leaf mass per area, 'LDMC'- leaf dry matter content, 'LA'-leaf area.

	Minor.VD	Major.VD	Major.MSTRatio	Minor.MSTRatio	FT	LMA	LDMC	LA
Minor.VD	1							
Major.VD	-0.44	1						
Major.MSTRatio	**0.56**	**-0.76**	1					
Minor.MSTRatio	**-0.70**	0.51	-0.49	1				
FT	-0.18	0.20	0.14	0.11	1			
LMA	**-0.80**	0.18	-0.30	**0.58**	0.12	1		
LDMC	0.41	-0.32	0.20	0.26	0.48	**0.73**	1	
LA	0.11	-0.31	0.02	0.27	-0.13	-0.31	-0.38	1

Table S2. Sequence of gas exchange measurements.

Date	Time of Day	Plants measured (in temporal sequence)
21 June 2018	8am-9am	*Prosopis velutina*-Plant 1<br>*Chilopsis linearis* – Plant 1
	9am-10am	*Chilopsis linearis* – Plant 1<br>*Rhus ovata* – Plant 1<br>*Vauquelinia californica* – Plant 1
	10am-11am	*Vauquelinia californica* – Plant 1<br>*Rhus ovata – Plant 2*
	11am-12pm	*Vauquelinia californica* – Plant 2<br>*Simmondsia chinensis* – Plant 1<br>*Simmondsia chinensis* – Plant 2
	12pm-1pm	*Simmondsia chinensis* – Plant 2<br>*Chilopsis linearis* – Plant 2
22 June 2018	8am-9am	*Olneya tesota* – Plant 1<br>*Olneya tesota* – Plant 2
	9am-10am	*Olneya tesota* – Plant 2<br>*Larrea tridentata* – Plant 1

	10am-11am	*Larrea tridentata* – Plant 2<br>*Populus fremontii* – Plant 1<br>*Populus fremontii* – Plant 2
	11am-12pm	*Prosopis velutina* – Plant 2<br>*Encelia farinosa* – Plant 1
	12pm-1pm	*Encelia farinosa* – Plant 2

DATA APPENDICES

Data files can be found as supporting information at the publication's online page at AoB Plants.

Data S1: *Gas exchange*

- File name: Gasexchange-DataS1.xlsx
- Data description: Simplified raw gas exchange data retrieved from LI6800 portable photosynthesis system. File contains species, plant and leaf identification along with its respective photosynthesis, transpiration and stomatal conductance measurements.

Data S2: *Leaf traits*

- File name: Leaftraits_proc-DataS2.xlsx
- Data description: Full dataset of average leaf traits per plant individual. Values reported are averages from leaves collected per plant (number of leaves varies per species, and compound leaves are based on all leaflets (i.e., whole leaf)), and includes raw leaf fresh and dry weights, leaf surface area, and estimated leaf dry mass content (LDMC), leaf mass per area (LMA), specific leaf area (SLA).

Data S3: *Force-to-tear*

- File name: Forcetotear_raw-DataS3.xlsx
- Data description: Raw force-to-tear estimates per leaf per species.

Data S4: *Minor leaf venation parameters*

- File name: Venation_raw-DataS4.xlsx
- Data description: Full dataset of leaf venation parameters (areoles and vein density) per leaf sample per species. Values reported are outputs from MATLAB script (https://github.com/bblonder/venation_programs) using partial leaf samples (1.5 x 1.5 cm).

Data S5: *Major leaf venation parameters*

- File name: MajorVenation_raw-DataS5.xlsx
- Data description: Full dataset of major vein parameters (vein width of 0.08 mm), vein density and mean spanning tree ratios (MSTs). Values reported are outputs from the LeafVeinCNN machine learning algorithm (https://ora.ox.ac.uk/objects/uuid:148dab65-3b83-4581-a757-21c19b5a9730) using whole-leaf samples.
